# Review and update on drugs related to the development of osteonecrosis of the jaw

**DOI:** 10.4317/medoral.23191

**Published:** 2019-12-24

**Authors:** Asier Eguia, Leticia Bagan, Francisco Cardona

**Affiliations:** 1 DDS, PhD. Associate professor. Universidad del País Vasco UPV/EHU. Leioa -Vizcaya, Spain; 2 DDS, MsC, PhD. Associate professor. Universitat de Valencia. Valencia, Spain; 3 MD, DDS, PhD. Head of Bucodental Health Section. Health Service of Navarra-Osasunbidea. Pamplona, Spain

## Abstract

**Background:**

Medication-related osteonecrosis of the jaw (MRONJ) is a rare, but serious adverse effect of certain drugs, of which bisphosphonates are the most widely known. This pathology is also associated with other medications such as the biologic antiresorptive agent, denosumab and some antiangiogenics such as sunitinib, bevacizumab or aflibercept. Very recently, new medications have also been associated with osteonecrosis of the jaw (ONJ). The objectives were to update the list of medications associated with ONJ, to analyze the fundamental aspects of this list and to describe the level of evidence available.

**Material and Methods:**

A narrative bibliographic review was made, using the PubMed-MedLine, DOAJ and SCIELO databases. Additional information was obtained through the online Medication Information Centre of the Spanish Agency of Medicines and Medical Devices (AEMPS – CIMA), the websites of the US Food & Drugs Administration (Drugs@FDA) and the European Medicines Agency (EMA).

**Results:**

The latest drugs identified as potential facilitators of this pathology include a number of anti-VEGF based antiangiogenic drugs and anti-TKI and different types of immunomodulators. Neither the level of evidence in this association nor the risk are equal for all these drugs. On the other hand, over the coming years, new drugs will be marketed with similar action mechanisms to those that are recognized as having this adverse effect.

**Conclusions:**

No effective therapy is currently known for the treatment of ONJ. Therefore, in order to prevent new cases of MRONJ, it is essential for all oral healthcare professionals to be fully up-to-date with the etiopathogenic aspects of this pathology and to be aware of those drugs considered to be a risk.

** Key words:**Osteonecrosis of the jaw, MRONJ, bisphosphonates, antiresorptives, antiangiogenics.

## Introduction

Osteonecrosis of the jaw (ONJ) is a rare, but serious pathology and can affect both jaws, although it is more common in the mandible. It manifests itself as one or more necrotic bone lesions, generally exposed in the oral cavity and which persist for at least 8 weeks (1-4). Numerous proposals (5-17) have been put forward with regard to the staging of ONJ as can be seen in Table 1.

**Table 1:**
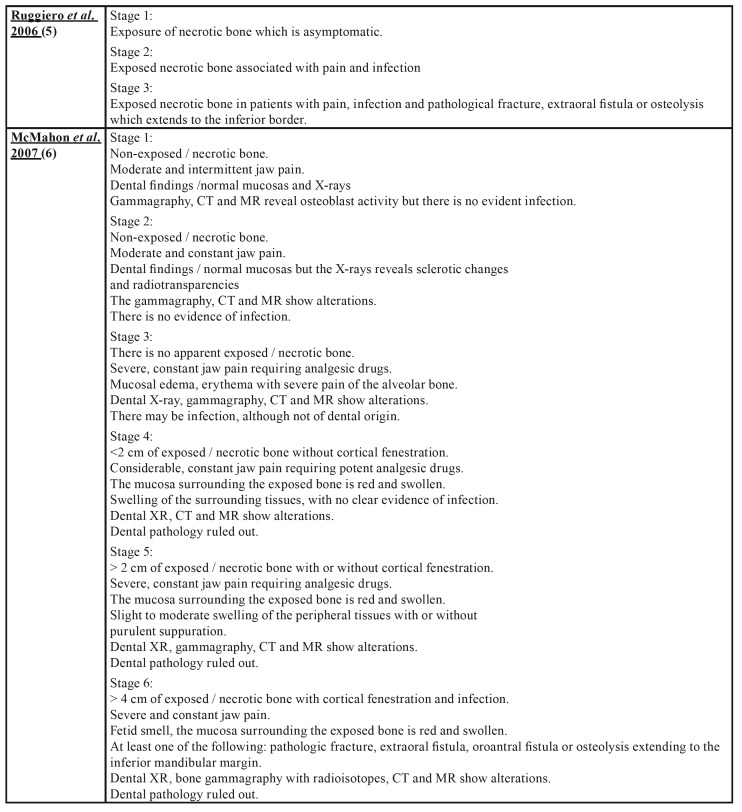
Osteonecrosis of the Jaw Staging Proposal by drugs.

**Table 1 cont.**
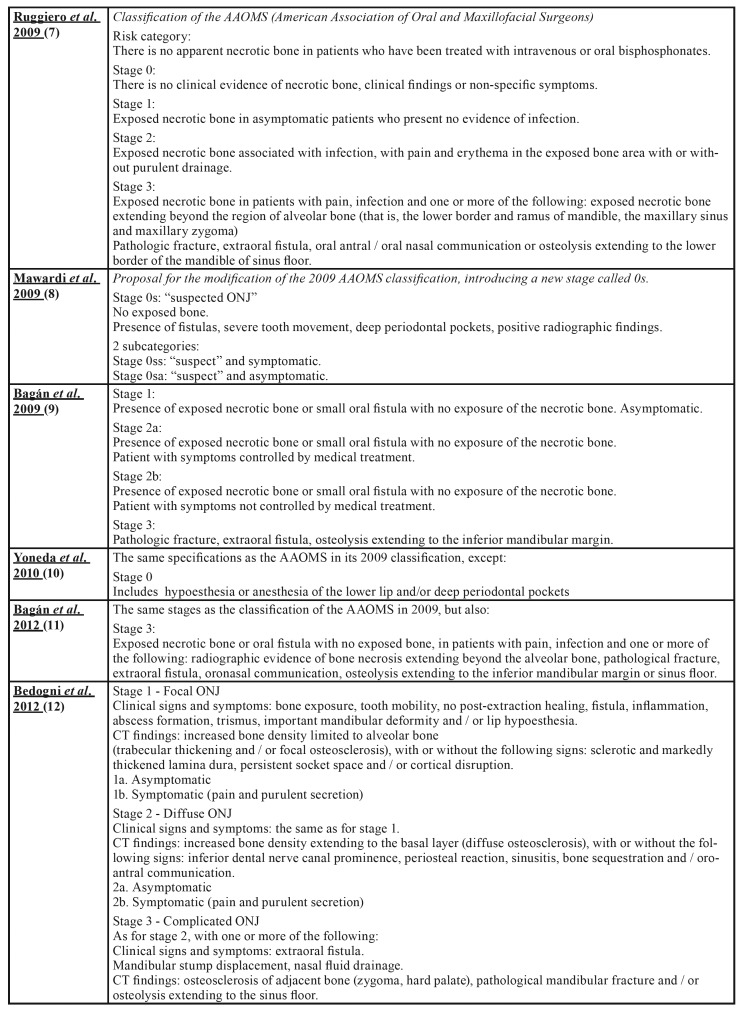
Osteonecrosis of the Jaw Staging Proposal by drugs.

**Table 1 cont.**
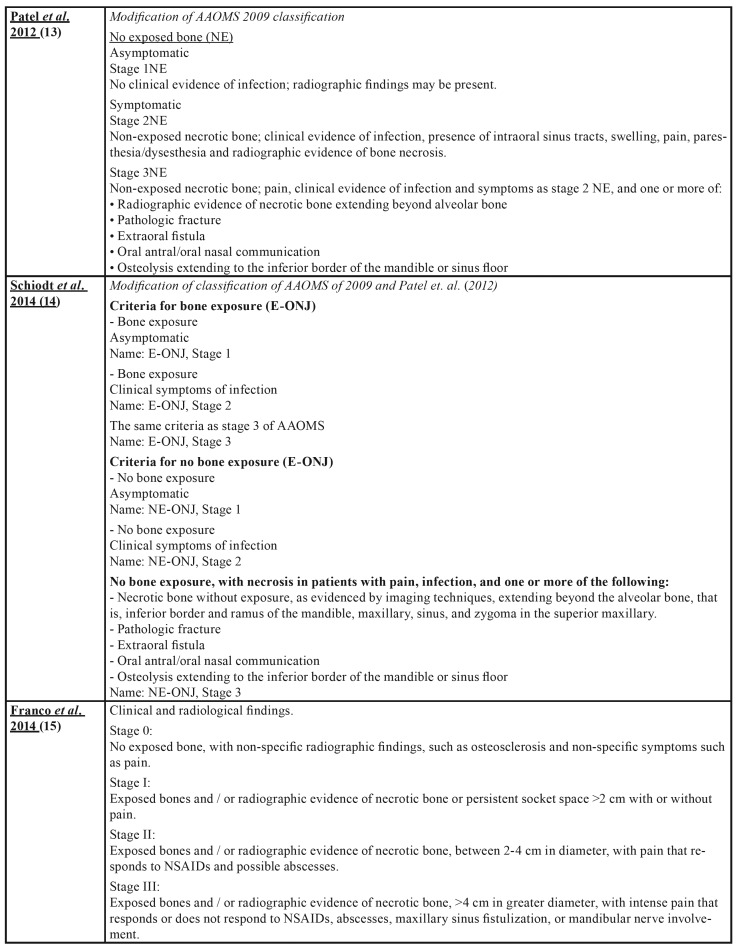
Osteonecrosis of the Jaw Staging Proposal by drugs.

**Table 1 cont.**
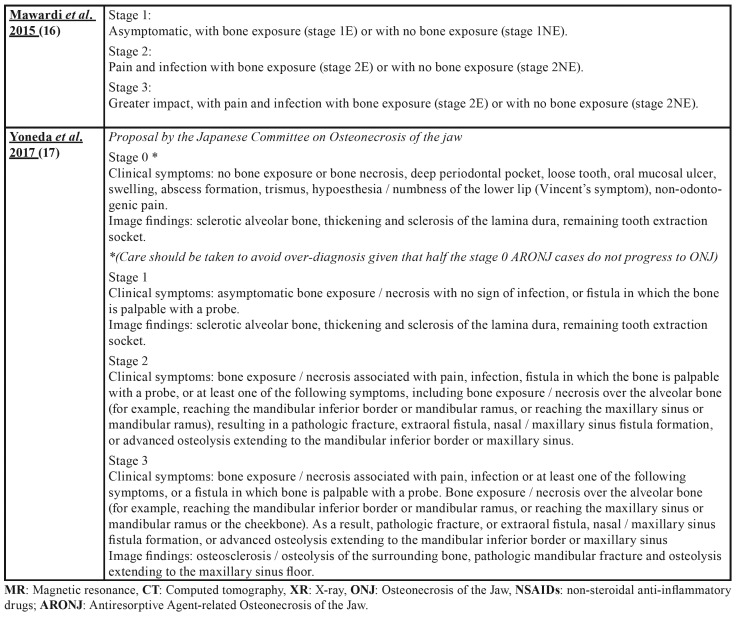
Osteonecrosis of the Jaw Staging Proposal by drugs.

**Figure 1:**
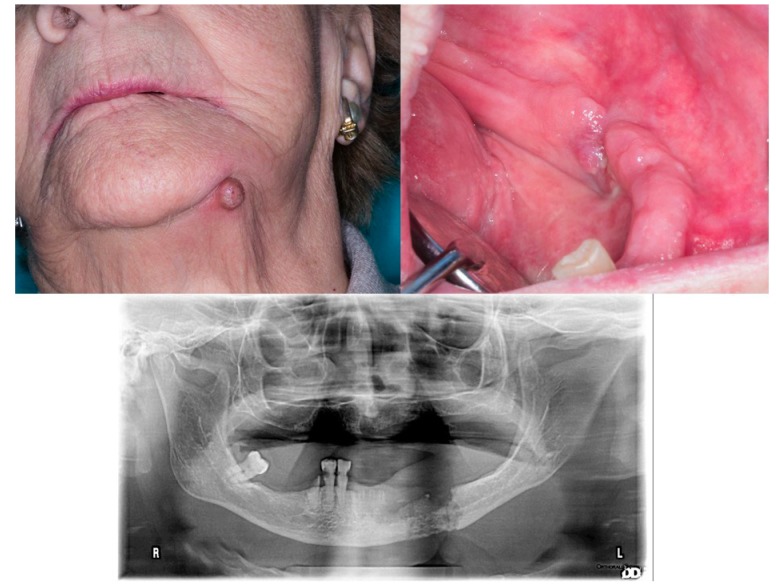
92-year-old woman. Treated with risedronate for five years due to spinal compression. History of tooth removal on the left side of mandible 4 months earlier. Exposed bone at the lingual side of lower left premolars, suppuration at this level (A) and a cutaneous fistula (B). C: The OPG shows extensive affected bone and mandibular fracture (not displaced).

It may be accompanied by pain, inflammation, loose teeth, erythema and suppuration. Although ONJ may occur spontaneously, in most cases it is a result of bone surgery: a tooth extraction or implant surgery, in patients who, prior to or immediately afterwards, have received pharmacological treatment with bisphosphonates, antiresorptive biologic agents or other medications detailed herein (1-4,18). Fig. 1.

ONJ has a long history, dating back to the end of the 19th century, when it was first described using the term "phossy jaw" for workers (primarily women) in match making factories. These factories used white or yellow phosphorous in the manufacture of matches, prior to the Berne convention in 1906, which limited its use. This raw material was highly toxic and contained pyrophosphate that was inhaled by the workers, leading to the appearance of ONJ as well as other serious diseases (19,20).

In 2003, R.E. Marx (1) published an article in which, for the first time, the appearance of 36 cases of ONJ was associated with the use of intravenous bisphosphonates (zoledronate and pamidronate) in patients with multiple myeloma or metastatic breast cancer. From then onwards, numerous cases of ONJ associated with the use of systemically and orally administered bisphosphonates have been published (1-4,18). Today, this relationship between bisphosphonates and ONJ is well-known, and a number of entities and associations have drafted guidelines and protocols for the prevention and treatment of this pathology (21-23).

Initially, the term BRONJ (Bisphosphonate Related OsteoNecrosis of the Jaws) was established to name this potential adverse effect (24). However, with the discovery that other medications such as the anti-RANK biologic antiresorptive agent (denosumab) (25) or the anti-VEGF antiangiogenic agent (bevacizumab) (26) and the TKI inhibitor (sunitinib) (27) could also be related to ONJ, from 2014 onwards, the term BRONJ was progressively replaced with MRONJ (Medication Related OsteoNecrosis of the Jaws) on the recommendation of the American Association of Oral and Maxillofacial Surgeons (AAOMS) (21).

At present, there is a growing list of drugs that could potentially cause ONJ, with varying levels of evidence. Although the risk of this serious adverse effect occurring varies considerably from one medication to another, it is also dependent on factors such as administration guidelines and dosage, length of treatment and the existence of concomitant systemic pathologies (4,18,21,22). In view of the imminent marketing of a number of biosimilar drugs and new biologic drugs, it is logical to foresee the continued growth of the list of ONJ-related drugs over the coming years.

Currently, there is no effective treatment for MRONJ (28) and therefore prevention is essential. This paper aims to review fundamental aspects of this pathology, to update the list of drugs that are potentially related to this adverse effect, and also to review the available evidence and the level of risk of the latest medications identified in the literature. The purpose is to ensure that all healthcare professionals are aware of the possibility of the occurrence of this pathology and to remind them which medications require the application of the MRONJ prevention guidelines and protocols.

## Material and Methods

A bibliographic review was made, using the PubMed-MedLine, DOAJ and SCIELO databases and different combinations of the terms included in the Medical Subject Headings (MeSH) of the Index Medicus/Medline: “osteonecrosis”, “jaw”, “Bisphosphonate-Associated Osteonecrosis of the Jaw”, “antiresorptive drugs”, “angiogenesis inhibitors” and “immunosuppressive agents”. Additionally, to complete the search, the following terms were employed: “antiangiogenic”, “drug-related”, “drug-induced”, “MRONJ”, “ARONJ”, “ONJ”, “BRONJ” and “antiresorptive”. Additional information sources were also used: the CIMA application (online Medication Information Centre) of the Spanish Agency of Medicines and Medical Devices (AEMPS), the websites of the US Food & Drugs Administration (Drugs@FDA) and the European Medicines Agency (EMA).

## Results

The medications related to MRONJ cases in the scientific literature were classified by pharmacological groups, as indicated below:

- Bisphosphonates

Bisphosphonates are used in the treatment of some skeletal dysplasias such as osteogenesis imperfecta or Paget's disease, osteoporosis and the prevention of hypercalcemia and bone events associated with bone metastases (1-4,18). Intravenously, they are primarily used in patients suffering from solid tumor bone metastases such as breast, prostate, bladder, lung and kidney and some lymphoproliferative processes (1-4,18). By contrast, bisphosphonates are primarily orally administered to patients suffering from osteoporosis. These drugs are able to bind to the bone matrix and, once released during bone resorption, they are able to induce the apoptosis of the osteoclasts (29). Due to this binding capacity, their effects can last for up to 10 years after treatment discontinuation (21). Although its mechanism of action is known, its relationship with the ONJ etiopathogenesis is only partially known (4,18).

The risk of ONJ in patients taking bisphosphonates depends on a number of factors, being significantly greater in: patients receiving intravenous administration, those accumulating a higher dose and for a longer period of time, those being administered corticoids concomitantly or suffering from other systemic pathologies such as diabetes or inflammatory joint pathology (4,18,21). Tobacco is probably also a negative influence, given its capacity to produce changes in the oral epithelium, to delay wound healing and to worsen the periodontal pathology (30). The relative potency of bisphosphonate is another factor that has been related to the risk of ONJ (31). "Non-nitrogenated" bisphosphonates such as etidronate and clodronate have the lowest potency and, therefore, offer a lower risk. They are principally used in rare bone dysplasias and dystrophies such as Paget's disease (31). Risedronate, ibandronate and alendronate are orally administered and are primarily used in patients with osteoporosis. Their relative potency is 10 to 100 times higher than the ones mentioned above and the prevalence of ONJ among patients is estimated at between 0.1% (10 cases/10,000) and 0.21% (21 cases/10,000), with the risk significantly increasing from the fourth year of treatment onwards (21,32). Pamidronate and zoledronate have a potency that is between 100 to 1000 times higher than the non-nitrogenated bisphosphonates and are administered intravenously, primarily in cancer patients. These are the ones at greatest risk and incidence data have been published with a variation between 0.7% and 6.7% of patients (21,33,34). In smaller prospective studies, as far as the number of patients is concerned, prevalence Figures of over 23% have been published (35).

The risk of ONJ in cancer patients not treated with antiresorptives, such as those assigned to the placebo group in controlled clinical trials, varies between 0% and 0.019%. Analyzing this and other available data, it can be seen that the risk of ONJ in cancer patients treated with zoledronate is between 50 to 400 times greater than for those not receiving antiresorptives (36,37).

Table 2 shows the list of bisphosphonates currently on the market, together with their brand name and route of administration.

**Table 2:**
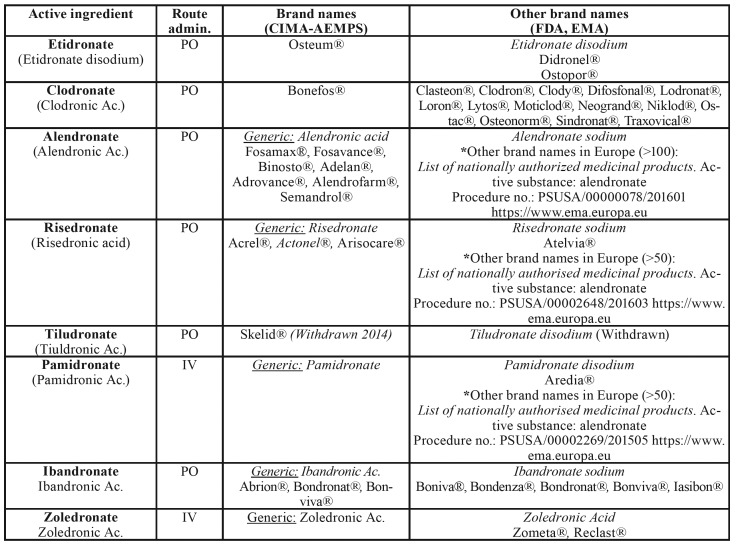
Bisphosphonates marketed in 2019. AEMPS – CIMA: Spanish Agency of Medicines and Medical Devices, Drugs@FDA: Archives of the US Food & Drugs Administration, EMA: European Medicines Agency.

- Biologic antiresorptives

Currently, denosumab (Prolia®, Xgeva®) is the main biologic antiresorptive associated with MRONJ (18,19,21,35). Denosumab is a humanized monoclonal IgG2 antibody that selectively binds to the ligand of the Receptor Activator of Nuclear Factor κβ (anti-RANKL) interfering in the system that regulates the bone metabolism, RANKL/RANK/OPG, emulating the biological effect of osteoprotegerin (OPG) (38). This selective blocking of the RANK ligand inhibits the activity and reduces the survival of the osteoclasts, resulting in a reduction in bone resorption and increased bone density (38). Unlike bisphosphonates, denosumab does not permanently bind to the bone matrix and, therefore, the residual effect on the remodeled bone could be minimal after 12-24 months after treatment cessation (21,38,39). However, the number of studies on the residual effects of denosumab following discontinuation are limited and with only a small number of patients (39,40).

**Figure 2:**
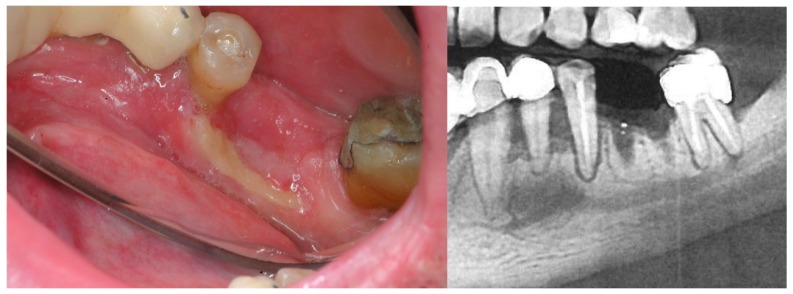
50-year-old male, HIV+, in treatment for depression and osteoporosis with denosumab (Prolia) for two years. He presented to the clinic because four months ago a lower molar was extracted and it does not heal. The patient was asymptomatic. B: Detail of the OPG showing poor ossification.

Denosumab is suiTable for the treatment of osteoporosis and the prevention of skeletal-related events (pathologic fracture, spinal cord compression, hypercalcemia) in adults with solid tumor bone metastases (28-40). In 2010, shortly after it was clinically introduced, the first cases of osteonecrosis related to its use, started to be published (25). Initially, the first cases were described for patients that had previously been administered bisphosphonates, however, subsequently, numerous cases have been described of patients that have not been treated with bisphosphonates and patients that were not given denosumab for an oncology process (38-40). The pathogenic mechanism that could facilitate the appearance of ONJ is only partially known and presents some differences of interest compared to bisphosphonates, both with regard to its antiangiogenic side effects and its immunomodulatory effects (41). In clinical terms, the lesions show no difference to those associated with bisphosphonates, however in some cases they could have a better response to treatment (40). Fig. 2.

 Today, the risk of ONJ in cancer patients under treatment with denosumab is estimated to be comparable to the risk of patients treated with zoledronate, and is estimated to be between 0.7% and 1.9% (21,28-41). With regard to patients treated for osteoporosis, the risk could be far lower, estimating it to be around 0.04% (42).

- Antiangiogenics: Anti-VEGF and Anti-TKIs

Antiangiogenics are substances of a different nature, capable of inhibiting the formation of new vessels. Due to this capacity, they are principally employed in oncology, given that neoangiogenesis is an essential process for the growth of tumors and the development of metastasis in some solid tumors (43). Of the numerous group of antiangiogenic drugs, the anti-VEGF (Vascular Endothelial Growth Factor Inhibitors) and the anti-TKI (Tyrosine Kinase Inhibitors) appear to be related to a greater risk of causing MRONJ (18,19,23). The precise etiopathogenesis of the ONJ associated with antiangiogenic drugs is not well know although, logically, the inhibition of angiogenesis negatively affects the bone regeneration capacity following bone aggression, delaying remodeling, healing and even increasing susceptibility to superinfection (44,45). Furthermore, these drugs have other effects. For example, bevacizumab and other anti-VEGF can inhibit macrophage chemotaxis and osteoblast differentiation (46) and sunitinib and other anti-TKIs can inhibit osteoclast differentiation and other cells of the monocyte / macrophage system, conditioning the local response of the immune system (47).

In 2008, the first cases of ONJ were published for cancer patients under treatment with bevacizumab (26), in 2009 with sunitinib (27) and subsequently in 2016 with aflibercept (48). Initially, some of the first clinical cases had previous or simultaneously received bisphosphonates and other risk medications. However, shortly afterwards, dozens of new cases started to be published for patients solely treated with these medications (4,18,44).

The last 5 years have also seen the publication of new cases of MRONJ associated with other antiangiogenics such as Dasatinib (49,50), Erlotinib (50), Imatinib (50,51), Axitinib (52), sorafenib (53) and cabozantinib (54). Therefore, these could be considered to be risk medications for MRONJ. However, for many of these medications, it has not been possible to quantify the said risk with a certain level of evidence, neither is the etiopathogenic relationship fully known. Moreover, there is no solid evidence to determine the exact duration of the said risk following treatment discontinuation.

The ONJ cases associated with antiangiogenic drugs may appear spontaneously or following surgery and, as is the case with antiresorptives, they are also more common in the mandible. The time between the start of treatment and the appearance of ONJ is extremely variable, ranging from a few weeks to 15 months (45,49-55).

Table 3 gives a list of the antiangiogenic drugs that have been related to cases of MRONJ, together with their brand name and route of administration.

- Biologic immunomodulators

Biologic immunomodulators are medications, generally humanized monoclonal antibodies, specifically designed to selectively bind to one of the inflammatory response mediators (56,57). Their inclusion in the battery of therapeutic products available has led to a tremendous advance in quality of life and a reduction in the severe side effects of other treatment alternatives for patients suffering from Crohn's disease, rheumatoid arthritis, ulcerative colitis, ankylosing spondylitis, or psoriatic arthritis among other diseases (56,57). As well as being indicated for this use, they are also useful in the treatment of some cancer processes. Very recently, isolated cases of ONJ have been described in the scientific literature, associated with the treatment of some of these medications such as infliximab (anti-TNFα) (58), adalimumab (anti-TNFα) (59,60) or rituximab (anti-CD20) (61,62).

**Table 3:**
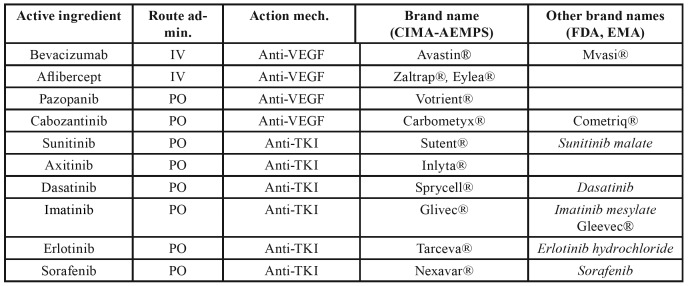
Antiangiogenics cited in the text: brand names, action mechanism and route of administration.

At present, there is no solid evidence to consider these as risk medications in relation to ONJ, given that there are no data to demonstrate a statistically significant greater incidence than in other population groups. Furthermore, there is no objective risk estimation that could potentially be used, and there is little knowledge on the etiopathogenic mechanism that could cause this adverse effect. In any case, considering the possibility of new cases appearing in the future and of being able to more definitely establish this association, it would be advisable to treat these patients with prevention protocols similar to the ones used for those taking antiresorptives or antiangiogenics.

The mechanism by which these drugs could increase the risk of ONJ has not yet been established. In the case of the anti-TNFα, their use could produce the inhibition of the bone remodeling at the cost of an inhibition of RANKL, with apoptosis induced by activated monocytes, or they could facilitate bone colonization by certain micro-organisms as a result of local immunosuppression (58-60,63). Table 4 lists the biologic immunomodulators for which cases of ONJ have been described, their brand name and the names of biosimilar products currently on the market.

- Other Immunomodulators

Corticoids: The long-term use of corticoids by the systemic route increases the risk of suffering osteonecrosis or avascular necrosis (AVN) (64,65). AVN involves the death of bone tissue and its marrow due to impairment of the blood supply to the bone tissue. Its most common sites are the femur, tibia, humerus, calcaneus, or scaphoid (64,65). At an oral level, corticoids do not appear to be capable of causing MRONJ by themselves, although patients concomitantly administered bisphosphonates or denosumab could have an increased risk of developing it (21,66,67).

**Table 4:**
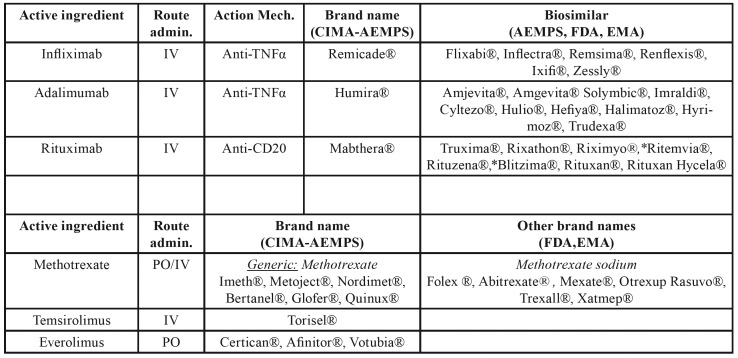
Immunomodulators cited in the text: brand names and route of administration.

Methotrexate: Methotrexate (MTX) is one of the key drugs used in the treatment of rheumatoid arthritis (RA) and other autoimmune disorders and inflammatory diseases (68). Furthermore, it is a cytotoxic medication that is indicated in the treatment of a number of solid tumors and haematological malignancies (68,69). The association of MTX with ONJ has some controversial aspects. Thus, in many cases described in the literature, in addition to MTX, patients had also been treated previously or concomitantly with bisphosphonates and/or corticoids (70). On the other hand, in some cases, the presence of necrotic bone could be secondary to a Methotrexate-related chronic lymphoproliferative disorder and it would therefore be arguable whether it should be considered as MRONJ, in the absence of a detailed histopathological study of the lesion (71). However, authors such as Henien *et al*. (72), have recently described cases of MRONJ in patients treated for RA with low oral doses of MTX over long periods of time, in the absence of a lymphoproliferative disorder and with no administration of other antiresorptive or antiangiogenic medications. The exact etiopathogenic mechanism of its possible association with MRONJ is unknown, although it appears to be primarily associated with its immunomodulating effects. MTX inhibits DNA synthesis, cell proliferation in the S phase cell cycle, it is cytotoxic in high doses while in low doses in inhibits T and B lymphocyte functions and proliferation, and inhibits the release of IL-1, TNFα and other cytokines (73,74). However, it could also be partly related to its great capacity to inhibit osteoblast proliferation (73,74).

mTOR inhibitors: Everolimus and Temsirolimus are inhibitors of mTOR (mammalian target of rapamycin) with antiangiogenic and immunosuppressive properties (75,76). They are primarily used to prevent transplant rejection and, at higher doses, they are used in the treatment of advanced stages of breast and kidney cancer, some neuroendocrine tumors and some types of leukemia (75,76). Most of the MRONJ related to Everolimus or Temsirolimus have been described in patients simultaneously receiving bisphosphonates (77,78), denosumab (79) or other antiangiogenics (80,81). However, MRONJ cases have recently been described in patients solely treated with Everolimus (82). The MRONJ cases described in the literature and associated with the mTOR inhibitors, do not appear to have different clinical characteristics to those associated with other medications (77-82). Given the paucity of clinical cases described up to now, it is not possible to estimate the level of risk for these medications in relation to MRONJ.

Table 4 also provides a summary of the brand names of the non-biologic immunomodulators associated with MRONJ.

- Other medications

Isolated cases of MRONJ have been described for other drugs, although it has not yet been possible to completely clarify the etiopathogenesis for this possible relationship. These drugs include ipilimumab (Yervoy®), an anti-CTLA-4 monoclonal antibody indicated for the treatment of advanced melanoma (83) or azacitidine (Vidaza®), a chemotherapy drug used in the treatment of some types of myelodysplastic syndromes and leukemia (84). At present, new investigations are required in order to ascertain whether these medications are related to a risk of MRONJ.

## Discussion

15 years after the first cases were reported relating to the development of ONJ with the use of bisphosphonates, there is still no effective treatment despite the different therapeutic options available (1,21-24). Prevention is key to controlling this severe pathology, which can have an important effect on a patient's quality of life (21-23). Its prevalence is extremely variable, depending on the drug, cause, duration and prescription dose, and the existence of other concomitant pathologies. The risk is low for those patients taking bisphosphonates or denosumab orally for osteoporosis and with no administration of corticoids and not suffering from other pathologies (21-23). However, this risk increases considerably from the fourth year of treatment onwards and is far higher in those patients receiving the intravenous administration of bisphosphonates. With regard to other medications, it is difficult to do a risk estimate due to the paucity of studies with a high level of evidence.

Although MRONJ may develop spontaneously, more than 60%-70% of cases, primarily dependent on the type of medication, occur following a dental extraction, implant surgery or any other type of jaw surgery (21-23). On occasions, even excessive pressure or a decubitus ulcer caused by removable dentures may trigger one of these lesions (21-23). Therefore, in view of the foregoing, in order to prevent MRONJ, it is essential for all healthcare professionals to have a thorough knowledge of the key clinicopathologic and etiopathogenic principles of this pathology.

Reviewing the literature, it can be observed how new drugs have been progressively added over the last few years to the list of drugs related to MRONJ, with a varying level of evidence. The patent of some biologic medications related to MRONJ has recently expired and, following approval, its biosimilar products have started to be used, although obviously to a lesser extent than the original products. Biosimilar drugs are not exact copies of the original biologic medication. However, they are designed to have the same biologic effect and, therefore, their possible adverse effects could also be very similar. Moreover, looking at the clinical trial registers such as ClinicalTrials.gov or EU Clinical Trials Registers, many more biosimilar medications related to MRONJ are already close to being marketed.

In view of the serious public health problem caused by MRONJ, it appears to be extremely important to ensure that all healthcare professionals are fully up-to-date with the complete list of risk medications, prevention protocols and consent documents prepared by the different entities and organizations in this field. Unfortunately, many of the documents available for consent, positioning and action protocols for this type of patient, are not up-to-date and do not include information on the drugs that have most recently come to be suspected of causing this side effect.

## Conclusions

 Not only bisphosphonates are capable of inducing ONJ. A growing list of medications probably have this same side effect, with a higher or lower risk. Although a high level of evidence does not exist for these medications, it would be important, from a preventive point of view, to apply clinical protocols that are the same or similar to the ones used for patients administered bisphosphonates or denosumab. Over the next 2 to 3 years, it would be advisable to treat with particular care those patients under treatment with new biologic antiresorptives and anti-inflammatory drugs, and any other new antiangiogenic or immunosuppressant.
